# Molecular Mechanisms and Therapies of Myeloid Leukaemia

**DOI:** 10.3390/ijms23116251

**Published:** 2022-06-02

**Authors:** Elliott Brown, Barbara-ann Guinn

**Affiliations:** Department of Biomedical Sciences, University of Hull, Hull HU6 7RX, UK; elliott.brown-2016@hull.ac.uk

Acute myeloid leukaemia (AML) is defined as a malignant disorder of the bone marrow (BM) that is characterised by the clonal expansion and differentiation arrest of myeloid progenitor cells [1]. It accounts for around 15–20% of leukaemias in the paediatric population and 5-year survival for paediatric and young adults is approximately 70% [2]. AML rates have been increasing over the last 40 years and this may reflect an aging population and exposure to more environmental factors, such as greater levels of pollution. Population effects such as the post-war ‘baby boom’ provided a large increase in the population reaching the age that is most associated with AML. In the last decade, this could account for some of the dramatic increase in AML diagnoses that was seen during the same period [3]. In the UK, AML is most often diagnosed between the ages of 75–84 (Figure 1) with only 1% of those affected being under 19 years of age.

The origins of AML are not exactly known; in adult patients, it is expected that the development of AML is due to the accumulation of genetic alterations, and this also predicts that the disease will be more complex due to the nature of its cause. With children, it is predicted that there is an in-utero mutation that leads to a predisposition to AML, while the development of AML occurs following a second genetic event at around 18 months of age (reviewed in [5]). Paediatric patients (from birth to 16 years of age) tend to respond a lot better to treatment as they often do not have the underlying health issues or comorbidities that are seen in adults which can complicate treatment [6]. However, the frequency of somatic mutations is greatly increased in adults with AML [7]. Notably, the mutational burden increases with age, with fusions and focal copy-number aberrations being more common in younger patients, whereas smaller sequence variants are more frequent in older individuals.

Treatment of paediatric AML patients primarily involves intensive chemotherapy, and for adults a program of induction, consolidation and maintenance chemotherapy. Induction therapy has changed little in the last 30 years and usually involves a combination of cytarabine and anthracyclines with recent trials showing low-risk paediatrics can safely receive fewer cycles of chemotherapy, in combination with haematopoietic stem cell transplant (HSCT) for high-risk patients with optimized infection and cardioprotective supportive care (recently reviewed in [8]). Siedlecka-Kroplewska et al. [9] demonstrated that piceatannol, a polyphenolic compound that is present in grapes, berries and passion fruit is a promising new chemotherapeutic agent for AML. The group used Piceatannol to induce caspase-dependent apoptosis in the AML M3 cell line, HL60. Although the cells could develop resistance via mechanisms related to MRP1 activity, this could be anticipated and circumvented through its use with other chemotherapeutic agents.

In adults, the use of HSCT is almost always the most effective treatment; however, in the low-risk subgroup of paediatric patients, HSCT was shown to have no effect on survival compared to multiagent chemotherapy [10]. Paediatric patients with a high risk of relapse (such as those with M0, M6 or M7) should have a non-familial HSCT while paediatrics with myelodysplastic syndrome (MDS) or secondary AML should receive HSCT from a sibling or matched donor [11]. The risk of graft versus host disease (GVHD) is notably lower in paediatrics than adults. As older patients tend to have other underlying health conditions that can complicate treatment, or they cannot tolerate more intense chemotherapeutic regimes, this can/does lead to elevated mortality rates in adults with AML. If a high-risk subgroup patient has an intra-family donor then an HSCT should always be performed to improve survival rates [11].

Targeted treatments are becoming more common, and the main benefit is that they are more specific to cancer cells, as opposed to conventional treatments that can also damage healthy rapidly dividing cells in the patient. One type of targeted therapy that has been used clinically since 2005 is monoclonal antibodies (mAbs) that bind to proteins on the surface of cancer cells [12]. Targeted therapies can be used to block growth, such as midostaurin (FLT3 inhibitor) [13]; encourage maturation with isocitrate dehydrogenase (IDH) inhibitors [14]; and/or lead to cell death. Such mAb therapies include gemtuzumab ozogamicin [15], an anti-CD33 binding mAb that induces cell-cycle arrest and induces apoptosis, or glasdegib that binds the transmembrane protein and targets the hedgehog pathway [16]. The main benefit to mAbs and other targeted treatments is the high specificity for the target cell, leading to less toxicity in non-cancerous cells [17]. The ideal AML treatment would have to have a high specificity for blasts and would not be present or would be present in considerably lower levels on normal stem cells, circumventing the death of healthy cells.

Immunotherapy, which includes activating the body’s own immune system to target and eliminate cancer cells is another promising treatment option. One method uses T-cells, and whilst this can produce the desired effects of killing leukaemic cells [18], T-cells can also cause harmful effects to the host’s healthy cells, such as cytokine release syndrome [19]. Immunotherapy covers a wide range of novel treatments (Table 1).

**Table 1:** Immunotherapy strategies.

Approach	Targets
Adoptive T cell therapy	CAR-T cells [20], CD44v6 CAR-T cells [21], FLT-3 CAR-T cells [22], CLL1/CD33 CAR-T cells [23]
Adoptive NK cell therapy	Infusion of haploidentical NK cells [24] and NK cell activation [25].
T cell engagers	Bispecific antibodies targeting CD33, tandem diabodies IL-3 and CD123 [26], BiTes and BiKes [27], dual affinity retargeting antibodies [28].
Checkpoint blockade and macrophage checkpoint blockade	PD-1 inhibitor [29], TIM-3 [30], CD47 [31].
mAbs	CD33, CD123, CD16, CD38, CD47, CD25 [32].
Vaccines	Boost immunity and provoke a response [33], Wilms’ tumour 1 (WT1) [34], P3 peptide vaccination [35].

Recently, treatments have included the use of chimeric antigen receptor T-cells (CAR-T) [36]. CAR-T cells have a recombinant receptor that can bind to antigens and kill targeted cancerous cells. The four components of the CAR itself are a hinge region, an antigen-binding domain, a transmembrane domain and a signalling domain [37]. Second generation CARs are in development that use tri-partite receptors comprising a costimulatory domain that can be used to target surface receptors. Additionally, CARs can bind to carbohydrates and glycolipid structures, as well as proteins. The CAR-T cells can also be combined with chimeric costimulatory receptors, cytokines or costimulatory ligands [38]. For CAR therapy to be successful in AML there needs to be exclusive AML surface antigens for them to bind to. Unfortunately, cancerous cells can develop resistance to single antigen targeting therapy. Despite initial promising results showing that CAR-T cells can target antigen-expressing cells effectively, some patients who have received CAR-T cell therapy show a subsequent absence of tumour antigen expression on their tumour cells [39]. Other obstacles that are related to CAR-T therapy include the challenges in getting the CAR-T cells into a solid tumour, further impeded by the tumour microenvironment and associated toxicity, including cytokine release syndrome and neurotoxicity [37].

GuinnLab studies antigens as potential targets for treatment and as biomarkers for disease diagnosis, prognosis and monitoring [40]. An ideal target for treatment would be an antigen that is expressed in most AML cells in most patient samples, as well as being expressed on the surface of the leukaemic cells and not expressed on healthy stem cells. Whilst a biomarker for prognosis needs to be expressed at considerably higher levels in diseased cells, compared with healthy cells, for it to be easily detectable, the existence of protein in healthy cells is not as crucial, as the cells will not be destroyed as part of the therapy. Previous research by the group has focused predominantly on adult AML, and for this reason, our recent work has aimed to evaluate the potential of some leukaemia-associated antigens that were found to be expressed in adults with AML (in this Special Issue [41]) and/or genes that are differentially expressed between risk subgroups in paediatrics and adults with AML [42].

A previous review highlighted the ideal cancer antigen characteristics, and these apply equally to leukaemias and solid tumours, although the target antigens often differ. To be useful as treatment targets for AML an antigen would need to be specific for leukaemia [43] and expressed by most AML cells in most patients. Cancer-testis antigens (CTAs), such as PASD1 and HAGE have the most potential as targets for therapy due to their specific expression in malignant cells; however, CTAs often have an expression that is limited to <35% of patients [44,45]. LAAs make promising targets and include SSX2IP, PRAME, WT1 and RHAMM but are not exclusively expressed in leukaemia cells (reviewed in [46]). The oncogenicity of antigens as targets for therapy should be considered along with their role within cancer cells and normal cell function. The immunogenicity of the antigen is critical as it is a specific immune response against the antigen, and in some cases this requires modifying the target epitope [47]. Preferably, immunotherapy would trigger the activation of cytotoxic T lymphocytes (CTLs) that are primed to kill the target cell. Finally, the clinical relevance of the antigen is important, the main objective being to provide benefit to the patient in the form of remission or a decrease in blast count.

In this issue, Greiner et al. [48] described the role of targeted immunotherapies in the treatment of AML—particularly, the removal of minimal residual disease in first remission to reduce the risk of relapse. In addition, allo-HSCTs rely on the replacement of the host stem cells by the incoming healthy donor stem cells. This is often boosted with donor leucocyte infusions to help support graft-versus-disease/graft-versus-leukaemia responses. Sadly, these often go hand-in-hand with graft-versus-host disease which can be life threatening. Other ways to support transplants could be the use of targeted immunotherapy to stimulate the donor immune response against the disease and facilitate engraftment. Greiner et al. explained how tumour antigens such as SSX2IP, survivin, PRAME, RHAMM and WT1 could be targeted. Indeed, in Greiner et al. [41], the authors demonstrate that above-median levels of survivin correlated with worse survival in inv(16) AML patients and that this could provide an immunologically relevant personalised target for a subset of AML patients.

Hindley et al. [49] reviewed the literature and discussed the importance of NPM1 gene mutations that are present in approximately 8% of paediatric AML patients and 25–30% of adults with AML. The authors described the structure and normal function of NPM1, the types of mutations and how this influences the progression of AML. Finally, the group described the importance of NPM1 mutations in predicting prognosis and the treatment options that are available to patients. The European Leukaemia Network (ELN) guidelines released in 2017 stratified risk into the categories of favourable, intermediate and adverse, depending on the genetic abnormalities present [50], and Hindley et al. explain why a rapid return of NPM1 mutational screening results can inform both prognosis and best practise treatment.

As we continue to search for new targets for therapy, our understanding of the role of post-translational modifications in the pathogenesis of disease grows. Vinney et al. [51] reviewed the role of deregulated epigenetics in myeloid malignancies, discussing how modifiers that regulate DNA methylation (i.e., DNMT3A, IDH1 and TET2) or histones (EZH2 and ASXL1) provide targetable mutations for a subset of AML patients. Mutations in genes that regulate DNA methylation (DNMT3A) or histone modification (ASXL1) have been shown to be associated with patient prognosis, demonstrating the need for therapies that target these high-risk aberrations. The global impact of these mutations on the genome needs to be better understood to ensure that the consequences of treatments targeting post-translational modifications are anticipated.

NUP98-rearrangements are enriched in paediatric AML patients who fail to achieve remission. These children are classified as having primary induction failure (PIF). Using 85 expression arrays and 358 RNAseq AML samples from the TARGET program, Barresi et al. [52] identified 110 differentially expressed genes (DEGs) when comparing NUP98+ and NUP98- patients. A qPCR confirmed nine DEGs in a local cohort of PIF patients that may act as biomarkers for PIF in AML and require further investigation.

Multiplex screening for interacting compounds in AML (MuSICAL) allowed all pairings of 384 FDA-approved compounds to be screened for their therapeutic potential while minimising cost, replication and redundancy. MuSICAL offered insights into an ‘untapped resource’ for the treatment of paediatric AML, particularly in the form of repurposing drugs that have already received FDA-approval. Of note, this study identified a triple combination of glimepiride, a sulfonylurea; pancuronium dibromide, a neuromuscular blocking agent; and vinblastine sulfate, a vinca alkaloid, as a potential therapy for paediatric AML patients [53] in the future.

Gorombei et al. [54] described a mouse model of high-risk myelodysplastic syndrome (HR-MDS) that reflects the human disease. The overall survival for patients with intermediate or higher risk MDS is poor, at only 0.8 years in the absence of treatment [55], and aggressive upfront approaches including allo-HSCT are warranted for medically fit patients. The HR-MDS mouse model was created with mutant NRAS and overexpressed BCL-2 [56], both prognostic indicators of the human disease. Gorombei et al. demonstrated that the BCL-2 inhibitor, ABT-737, significantly extended survival in the HR-MDS mouse with reductions in BM blasts and primitive progenitor cells [54], by targeting the leukaemia-initiating (or cancer stem cell) population. These mouse studies provide a three-pronged approach to fundamental questions. Namely, they demonstrate the application and optimization of personalized treatment protocols, supported by the in vivo analysis of resistant mechanisms to both generic and targeted therapies; they facilitate pathway analyses which allow the identification and interrogation of differentially regulated expression profiles that regulate key gene signatures; and finally, they provide stratification profiling data.

## Figures and Tables

**Figure 1:**
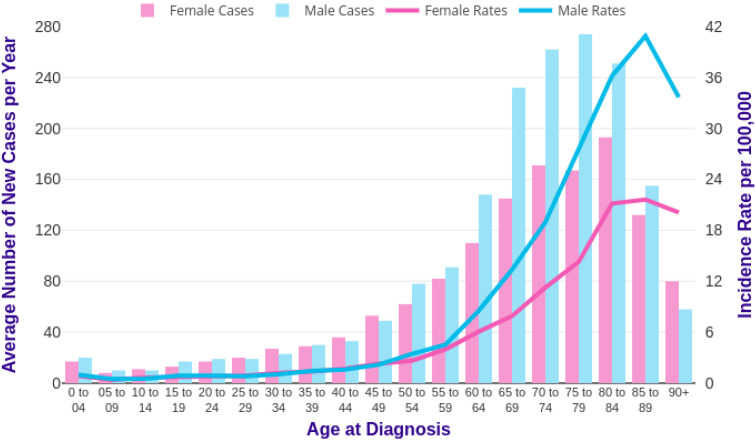
AML incidence in UK by age. A small increase in AML cases is shown between 0–4 years of age, followed by a steady increase post-10 years. Significant disparity between males and females appears from 60–64. Data from Cancer Research UK [4].

## Data Availability

Not applicable.

## Abbreviations

AML	Acute myeloid leukaemia
BM	Bone marrow
CAR	Chimeric antigen receptor
HSCT	Haematopoietic stem cell transplantation
IDH	isocitrate dehydrogenase
mAbs	monoclonal antibodies
